# Lumbar Spinal Fusion Outcomes in Patients With Cancer Compared to Matched Peers Without Cancer

**DOI:** 10.1177/21925682241307631

**Published:** 2024-12-05

**Authors:** Ryan S. Gallagher, Ritesh Karsalia, Emily Xu, Connor A. Wathen, Austin J. Borja, Jianbo Na, Tara Collier, Scott McClintock, Neil R. Malhotra

**Affiliations:** 1 Department of Neurosurgery, 14640 Perelman School of Medicine at the University of Pennsylvania, Philadelphia, PA, USA; 2 6572 McKenna EpiLog Fellowship in Population Health, at the University of Pennsylvania, Philadelphia, PA, USA; 3 The West Chester Statistical Institute and Department of Mathematics, 8510 West Chester University, West Chester, PA, USA

**Keywords:** lumbar fusion, cancer, malignancy, spine surgery, coarsened exact matching

## Abstract

**Study Design:**

Retrospective Matched Cohort Study.

**Objectives:**

Optimization of medical comorbidities is an essential part of preoperative management. However, the isolated effects of individual comorbidities have not been evaluated within a homogenous spine surgery population. This exact matching study aims to assess the independent effects of cancer on outcomes following single-level lumbar fusions for non-cancer surgery.

**Methods:**

4680 consecutive patients undergoing single-level posterior-only lumbar fusion were retrospectively enrolled. Univariate statistics and coarsened exact matching (CEM) were computed to evaluate outcomes between cancer patients and those without comorbidities.

**Results:**

By logistic regression, malignancy conferred a higher risk of surgical complication (*P* = 0.016, OR = 2.64, CI = [1.200,5.790]), 30- and 90- day readmission (*P* = 0.012, OR = 2.025, CI = [1.170-3.510]; *P* < 0.001, OR = 2.34, CI = [1.430, 3.830], respectively), 90-day reoperation (*P* < 0.001, OR = 2.16, [1.110, 4.200]), and death at 90-days (*P* = 0.032, OR = 8.27, CI = [1.200, 56.850]). After matching, malignancy was associated with increased odds of incidental durotomy (6 vs 0 cases, *P* = 0.048) and death at both 30 and 90 days (both: OR = 8.0, *P* = 0.020, CI = [1.00, 63.960]). No cases of durotomy occurred in cases with mortality in the matched sample, suggesting independent relationships. There were no differences in length of stay, non-home discharge, ED evaluation, readmission, or reoperations.

**Conclusion:**

Among otherwise exact-matched patients undergoing single level lumbar fusion, history of malignancy conferred a higher risk of short-term mortality, but not other outcomes suggestive of surgical failure. Increased mortality after lumbar fusion should be studied further and may play a role in surgical decision-making and patient discussions.

## Introduction

Lumbar fusion is a common and increasingly performed procedure worldwide.^1^ Baseline complication and mortality rates are low^2-4^ and studies suggest that the cost/quality-adjusted life years ratio of lumbar fusion may be preferable to that of conservative care for degenerative spine conditions.^5,6^ However, given the overall increase in cost burden to health systems from lumbar fusions, it remains necessary to optimize the safety and the cost-to-value ratio of this procedure.

Many patient-specific risk factors for markers of poor surgical outcomes are being studied to guide the selection for surgery and focus targets for perioperative management.^7-12^ Medical comorbidity is a common risk factor examined by such studies. The Charlson Comorbidity Index (CCI) and American Society of Anesthesiologists (ASA) Classification are composite risk scores of comorbidity and have been shown to be associated with adverse short-term outcomes from lumbar surgery.^7,8,12-14^ In addition to composite measures like the CCI or global assessments of disease severity like the ASA classification, individual comorbidities have also been studied, showing that malignancy, rheumatoid disease, diabetes, and asthma may independently relate to patient outcomes following spinal surgery.^12,15,16^

Malignancy of any kind has been repeatedly shown in nationwide databases to associate with increased reoperations and mortality after lumbar fusion surgeries.^17,18^ Furthermore, patients with cancer have several related conditions, such as poor functional status, electrolyte abnormalities, and hypercoagulability, that impact surgical outcomes.^19^ Despite this growing literature, databases are susceptible to errors^20^ and well-controlled single institution studies can provide external validity. As perioperative patient care pathways are becoming increasingly standardized, it is imperative to have a thorough understanding of patient-specific risk factors to refine perioperative medical management to meet each patient’s unique needs. Additionally, the trend towards value-based care places strong incentives on hospital management to develop risk mitigation pathways on a health systems-level. We aim to examine how a specific medical comorbidity is associated with outcomes and rigorously control for the complex interactions between demographic, medical, and surgery specific risk factors by matching patients undergoing a precisely defined surgical procedure. Given malignancy as a leading cause of mortality in western societies, we focus herein on history of malignancy as it directly relates to the odds of adverse surgical outcomes following single-level lumbar fusion.

## Methods

### Patient Selection

**Figure 1.**
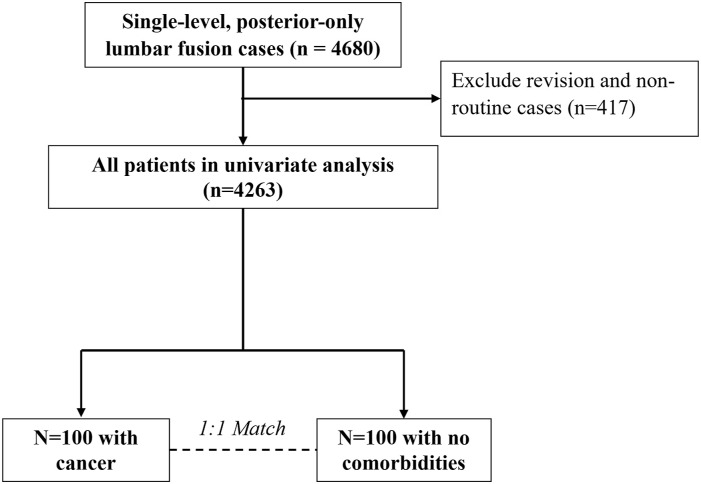
Study selection and sample size.

A total of 4680 consecutive cases of adult patients undergoing single-level posterior-only lumbar fusion at a single multihospital academic medical center from 2013 to 2021 were prospectively enrolled and retrospectively studied. The indications for spinal fusion in our cohort included symptomatic spondylolisthesis, radiculopathy, and degenerative scoliosis.^21^ Inclusion criteria included cases that were non-emergent, inpatient admissions, used general anesthesia, had clean wounds, and had complete follow-up information. A total of 4263 cases analyzed further (Figure 1).

### Data Extraction

Patient characteristics and surgical outcome data were extracted from the electronic medical record (EMR) using EpiLog, a non-proprietary system integrated with the EMR to streamline data collection and quality improvement initiatives.^22^ Patient variables controlled for included median household income (MHI) cross-referenced to zip-code (adjusted to 2016 US dollars), body mass index (BMI), age, sex, race, ASA score, smoking status, prior surgical history, insurance type (public vs private), duration of surgery, and presence of each comorbidity measured within the CCI score. Outcomes measured included surgical complication, length of stay, discharge home vs non-home, and 30- and 90- day emergency department (ED) evaluation, readmissions, reoperation, and all-cause mortality.

### Statistical Analysis

Patients with any malignancy (n = 121) were compared to patients with no medical comorbidities (n = 2329), as measured by all CCI variables excluding age. First, univariate logistic regression was performed to determine if malignancy correlated with short-term surgical outcomes. Next, coarsened exact matching (CEM) was employed to seek ideal matches on patient characteristic known to impact outcome, except malignancy. CEM was performed by binning the following 10 controlling variables into categorical levels: age (categorized by decade), ASA grade (exact matched), health insurance type (private vs public insurer), gender (male vs female), smoking history (prior smoker vs never user), prior surgical procedures (binary), prior surgery within 30 days of index operation (binary), BMI (<18.5, 18.5-30, or >30), MHI (above or below median), and duration of surgery (above or below median). Patients with malignancy were then matched 1:1 to those without any medical comorbidities (n = 200 matched patients). Patient demographics were compared using chi-squared and non-parametric tests. Outcomes in the CEM cohort were analyzed using the McNemar’s and non-parametric tests for categorical and continuous variables, respectively.

The SAS version 9.4 (SAS Institute Inc) program was used to bin covariates and remove missing values for CEM and subsequent matching done via the MatchIt programming package in R Statistics (R Core Team, 2017). All other statistical analyses were performed with SAS version 9.4. Significance for all analyses was set as *P*-value <0.05.

## Results

### Patient Demographics

**Figure 2.**
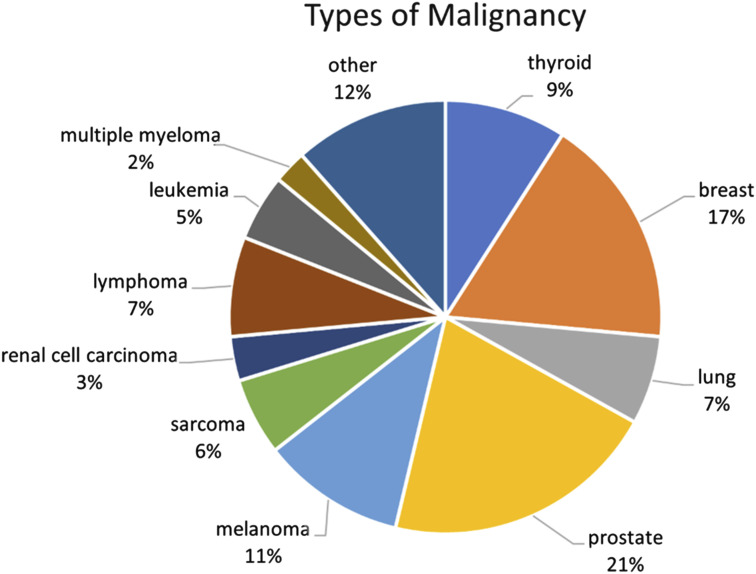
Representation of cancer type in malignancy cohort (n = 121).

Before matching, patients with malignancy (n = 121) tended to be older (66.50 vs 58.75, *P* < 0.001), smoke less (4.13% vs 14.30%, *P* = 0.0005), have higher ASA scores (2.48 vs 2.24, *P* < 0.0001), be publicly insured (64.5% vs 42.9%, *P* < 0.0001), and have more prior surgeries (0.92 vs 0.52, *P* = 0.0004) than patients without comorbidity (n = 2329). After matching, there were no statistically significant differences in age, tobacco use, insurance type, or surgical history between matched groups. Patient demographic information before and after matching is presented in Table 1 and breakdown of cancer types in the malignancy cohort is displayed in Figure 2.

**Table 1.** Demographics in the Cohort of Cancer Analysis.

	Before Matching			After Matching		
	No Comorbidities (n = 2329)	Cancer History (n = 121)	*P*-value	No Comorbidities (n = 100)	Cancer History (n = 100)	*P*-value
Gender (n, %)						
Male	1050 (45.08%)	403 (47.93%)	0.5745	48 (48.00%)	48 (48.00%)	1
Female	1279 (54.92%)	231 (50.55%)		52 (52.00%)	52 (52.00%)	
Age (mean, range)						
	58.75 [15.0,90.0]	66.50 [27.0,84.0]	<0.0001	65.98 [31.0,85.0]	66.42 [27.0,83.0]	0.5829
Race (n, %)						
White	1959 (84.11%)	105 (86.78%)	0.0849	91 (91.00%)	87 (87.00%)	0.1189
Non-White	370 (15.89%)	16 (13.22%)		9 (9.00%)	13 (13.00%)	
BMI (mean, range)						
	29.26 [15.0, 54.4]	30.50 [13.64, 56.58]	0.2957	28.92 [19.75, 54.4]	28.58 [18.6,41.75]	0.93
Tobacco use (n, %)						
	333 (14.30%)	5 (4.13%)	0.0005	2 (2.00%)	2 (2.00%)	1
Insurance type (n, %)						
Private	1329 (57.1%)	43 (35.5%)	<0.0001	35 (35.0%)	35 (35.0%)	1
Public	1000 (42.9%)	78 (64.5%)		65 (65.0%)	65 (65.0%)	
Prior surgery (mean, range)						
	0.52 [0,11]	0.92 [0,17]	0.0004	0.65 [0,7]	0.87 [0,17]	0.90
Prior surgery 30D (mean, range)						
	0.03 [0,3]	0.02 [0,1]	0.668	0.03 [0,3]	0.02 [0,1]	1
ASA score (mean, range)						
	2.24 [1.0,4.0]	2.48 [2.0,3.0]	<0.0001	2.45 [2.0,3.0]	2.45 [2.0,3.0]	1

### Statistical Analysis

**Figure 3.**
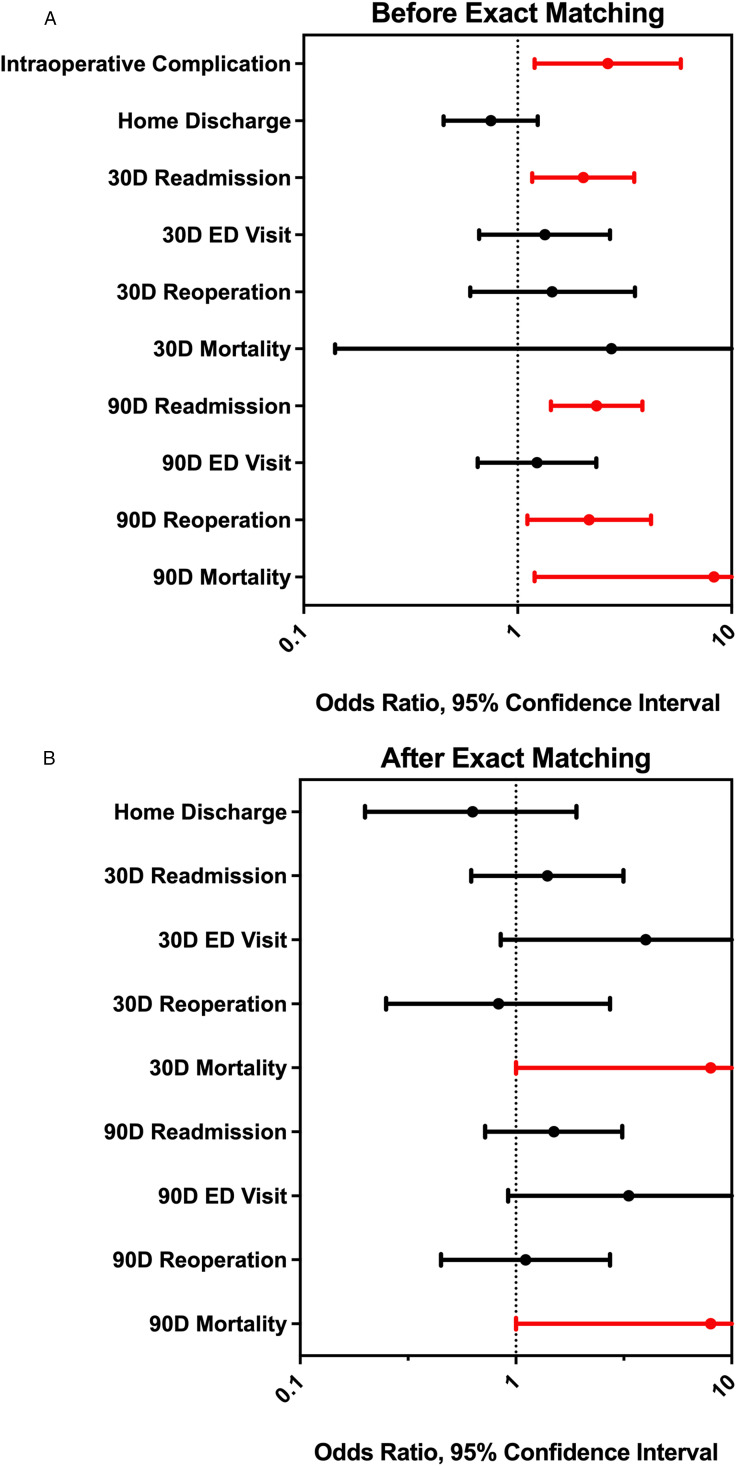
(A) Forest plot for univariate regression analysis showing odds ratios and 95% confidence intervals for postoperative outcomes. Significant values (*P*-value <0.05) are denoted in red. (B) Forest plot for coarsened exact matching analysis showing odds ratios and 95% confidence intervals for postoperative outcomes. Significant values (*P*-value <0.05) are denoted in red.

In the univariate regression model (Figure 3A), malignancy was associated with higher odds of incidental durotomy (*P* = 0.016, OR = 2.64, CI = [1.200,5.790]), 30- and 90- day readmission (*P* = 0.012, OR = 2.025, CI = [1.170-3.510]; *P* < 0.001, OR = 2.34, CI = [1.430, 3.830], respectively), 90-day reoperation (*P* < 0.001, OR = 2.16, CI = [1.110, 4.200]), and death at 90-days (*P* = 0.032, OR = 8.27, CI = [1.200, 56.850]). There were no differences in rates of non-home discharge, 30- or 90- day ED visits, 30-day reoperation rates, or 30-day mortality. After CEM, malignancy was found to be associated with significantly greater incidental durotomy (6 vs 0 cases, *P* = 0.048) and increased odds of death at both 30 and 90 days (both: OR = 8.0, *P* = 0.020, CI = [1.00, 63.960]) but no different length of stay, or odds of discharge home vs not home, ED evaluation, readmission, or reoperations (Figure 3B). No deaths occurred among the patients with incidental durotomy in the matched cohort.

## Discussion

This study examines how the presence or absence of any cancer history affects the short-term outcomes of non-cancer-related lumbar fusion surgery in a single-center cohort study. Univariate analysis showed that history of malignancy was related to increased odds of surgical complications, readmissions, reoperations, and death. After controlling for confounders by matching patients with malignancy to those without any medical comorbidities on 10 factors associated with outcomes, malignancy was associated with higher odds of incidental durotomy, and, unrelatedly, death, but not length of stay, discharge home vs non-home, or short-term ED visits, readmissions or reoperations. It has been shown previously that metastatic cancer and lymphoma are associated with complications, readmissions and mortality in multivariate modeling, and our finding is a novel addition to this literature providing an analysis exactly matching cancer patients to patients without medical comorbidities.^3,15,23^

It is likely expected that there are higher odds of death after surgery in patients with a history of malignancy compared to those without. Despite higher mortality rates, patients with cancer did not demonstrate any differences in length of stay, ED visits, readmissions, or repeat operations compared to otherwise exactly matched peers without malignancy. These findings may suggest that single-level lumbar fusion in well-selected patients, even with malignancy, can remain a safe and effective procedure, despite a higher baseline mortality rate from malignancy alone.

Cancer is the second leading cause of mortality in America and worldwide.^24,25^ While this study was conducted in a United States medical institution, our results provide relevant insights into the interaction between a common medical comorbidity and outcomes of a spine procedure that is widely conducted in other countries.^26-28^ Regardless of the specific healthcare system, it is important to understand how patient characteristics may impact surgical management and our study may provide groundwork for further analyses at international institutions.

The risks of major surgery such as lumbar fusion may be magnified in patients with malignancy due to the systemic effects of cancer on the body, irrespective of the cancer subtype. Patients with cancer share several of the same underlying risk factors, including greater rates of anemia, frailty, and are predisposed to medical complications such as deep vein thrombosis and pulmonary embolism.^29-32^ Blood loss is associated with greater morbidity and mortality during spine surgery, surgical/hospital practices on blood transfusions are variable and contribute to outcomes, and malignancy history may influence surgical decisions regarding transfusion.^33,34^ Furthermore, as a product of both the underlying disease process and the effects of treatment, cancer patients are subject to increased levels of immunosuppression relative to healthy peers.^35^ There are also several psychosocial factors and significant psychiatric comorbidities that are associated with having a life-threatening and chronic disease such as cancer.^36,37^ A combination of these baseline patient factors with the immunosuppressive effects of surgical trauma,^38^ the effect of blood loss on pre-existing anemia and frailty, and the increased risk of DVT and PE seen following spine surgery may all contribute to the observed increased mortality rate seen in these individuals in addition to the increased baseline risk.^39^

As a result of these risk factors, the perioperative management of cancer patients must be carefully tailored.^19^ After a potential patient is identified to have a history of malignancy, the surgeon should first assess whether they would still be a good surgical candidate given their comorbidity and its implications on life expectancy. The patient should also be informed of the additional risk, so that they can weigh it with the potential benefit of the intervention. Additionally, because cancer is associated with various acute and chronic pain syndromes,^40^ there should be consideration of the efficacy of surgery and whether the patient is willing to tolerate the postoperative pain. Once the decision to proceed with surgery is made, the patient should be engaged with other specialists to optimize their medical management prior to surgery, including comprehensive assessments of functional status, baseline cancer-related pain, and nutritional status. Furthermore, chemotherapy and/or radiation timelines should be considered when scheduling an elective surgery given their effects on wound healing.^41^ Patients should be optimized with regards to anticoagulation due to their increased hypercoagulability^39^ and perioperative antibiotics may need to be tailored given that many cancer patients are immunocompromised.^42^ Additionally, many cancer patients have adrenal insufficiency due to chronic steroid treatment^43^ and may therefore require increased dosing of glucocorticoids. Postoperatively, close follow-up should be coordinated with the patient’s oncologist and primary care provider to manage pain, wound healing complications, and psychosocial issues.^36,37^

In addition to the increased odds of mortality, we note that patients with cancer had increased incidental durotomy than those without cancer, although between matched patients there was no co-occurrence of durotomy and mortality. Notably, this study was performed on a cohort of patients undergoing lumbar fusion surgery for reasons other than spinal tumors. This is an important area for future investigation as understanding the role of a patient’s cancer status on dural integrity and surgical complications could facilitate adjustments to surgical technique or perioperative management to mitigate these occurrences.

### Limitations

This study comes with important limitations. The patients analyzed in this study were from a single multihospital academic medical center, potentially introducing bias. By design of the retrospective analysis, this study matched and compared within a cohort of patients selected for and undergoing surgery. Lacking a control group of similar patients with malignancy who did not undergo surgery, we cannot determine whether the observed mortality rate in this surgical cohort differs from the baseline mortality rate for malignancy alone. We are limited to observing that, for well-selected patients, higher odds of mortality did not coincide with any relationship to the length of stay, ED visits, readmissions, or reoperations. Additionally, we were unable to assess the specific reasons for increased mortality and further studies are warranted to understand the underlying causes. Another limitation is that the outcome variables, although selected for interpretability and importance to patients, providers, and payers, do not capture subjective patient outcomes. Finally, separating all patients with a history of malignancy together in our matching cohort from those with no comorbidities, while enabling a larger cohort by our matching method, likely oversimplifies the complex heterogeneity among all cancers. Informed by our results and the prior literature, ongoing and future studies should explore these nuances of cancer history, types, and staging may influence patient experiences of degenerative spine disease and functional surgical outcomes.

### Conclusion

Patients with a history of malignancy undergoing single level lumbar fusion have a higher short-term mortality risk compared to exact-matched patients without cancer. However, there is no significant difference in other measures of healthcare utilization indicating surgical differences. In this cohort, surgeons appear to deliver equally safe short-term immediate surgical outcomes to patients with or without malignancy; however, increased early death in patients with malignancy should lead to additional research and play a role in strategies to optimize care for cancer patients.

## Appendix

### Abbreviations

MHI: Median Household Income
ED: Emergency Department
OR: Odds Ratio
CEM: Coarsened exact matching
BMI: Body mass index
ASA: American Society of Anesthesiologists
CCI: Charlson Comorbidity Index
